# Participation in the Child and Adult Care Food Program is associated with fewer barriers to serving healthier foods in early care and education

**DOI:** 10.1186/s12889-020-08712-7

**Published:** 2020-06-05

**Authors:** Daniel A. Zaltz, Amelie A. Hecht, Russell R. Pate, Brian Neelon, Jennifer R. O’Neill, Sara E. Benjamin-Neelon

**Affiliations:** 1 Department of Health, Behavior and Society, Johns Hopkins Bloomberg School of Public Health, 615 N. Wolfe St, Baltimore, MD 21205 USA; 2 Department of Health Policy and Management, Johns Hopkins Bloomberg School of Public Health, 615 N. Wolfe St, Baltimore, MD 21205 USA; 3 Department of Exercise Science, University of South Carolina Arnold School of Public Health, 921 Assembly St, Columbia, SC 29208 USA; 4 Division of Biostatistics, Department of Public Health Sciences, Medical University of South Carolina, 135 Cannon St, Charleston, SC 29425 USA

**Keywords:** Barriers, CACFP, Child care, Healthy eating, Nutrition

## Abstract

**Background:**

Early care and education (ECE) is an important setting for influencing young children’s dietary intake. There are several factors associated with barriers to healthy eating in ECE, and recent evidence suggests that participation in the Child and Adult Care Food Program (CACFP), the primary national food assistance program in ECE, may be associated with fewer barriers to serving healthier foods. However, no prior studies have examined differences between CACFP participants and non-participants across a large, multi-state sample. This is the first study to examine the association between CACFP participation and barriers to serving healthier foods in ECE using a random sample of directors from two regions across the country.

**Methods:**

We conducted a cross-sectional survey among a random sample of child care center directors from four states (Massachusetts, North Carolina, Rhode Island, and South Carolina). We conducted logistic and Poisson regression to calculate the odds and incidence rate ratios of reporting 1) no barriers, 2) specific barriers (e.g., cost), and 3) the total number of barriers, by CACFP status, adjusting for covariates of interest.

**Results:**

We received 713 surveys (36% response rate). About half (55%) of centers participated in CACFP. The most prevalent reported barriers to serving healthier foods were cost (42%) and children’s food preferences (19%). Directors from CACFP centers were twice as likely to report no barriers, compared to directors from non-CACFP centers (OR 2.03; 95% CI [1.36, 3.04]; *p* < 0.01). Directors from CACFP centers were less likely to report cost as a barrier (OR = 0.46; 95% [CI 0.31, 0.67]; *p* < 0.001), and reported fewer barriers overall (IRR = 0.77; 95% CI [0.64, 0.92]; *p* < 0.01), compared to directors from non-CACFP centers.

**Conclusions:**

CACFP directors reported fewer barriers to serving healthier foods in child care centers. Still, cost and children’s food preferences are persistent barriers to serving healthier foods in ECE. Future research should evaluate characteristics of CACFP participation that may alleviate these barriers, and whether barriers emerge or persist following 2017 rule changes to CACFP nutrition standards.

## Background

Dietary habits developed during the preschool years may track into adulthood and impact lifetime risk of obesity [1–3]. Early care and education (ECE) settings are important venues for influencing young children’s dietary intake [4, 5]. More than half of American children aged 5 years or younger attend regular ECE [6], and current guidelines suggest providing children in full-time care with up to two-thirds of their daily caloric intake in ECE [7]. One way to influence young children’s diet quality in ECE is through the implementation of healthy eating policies [7–11], which often set age-appropriate limits to the types and amounts of less healthy foods that can be served, such as sugar-sweetened beverages or high-fat meats, and prescribe minimum amounts of healthier options to be served, like fruits, vegetables, and whole grains [9, 12–16].

Most healthy eating policies are set and enforced as requirements for licensure by state-level regulatory agencies [5, 8, 17]. However, a variety of federal agencies and organizations publish recommendations for healthy eating in ECE [10, 18–20] which may ultimately impact these state-level policies. For example, more than half of US states require all child care centers to follow Child and Adult Care Food Program (CACFP) nutrition standards, regardless of program participation [21]. The CACFP is the primary federal nutrition assistance program for ECE and serves over four million low-income children per year [22]. Participating ECE programs receive reimbursements for eligible meals, snacks, and beverages in compliance with the USDA nutrition standards [23]. Non-profit child care centers and family child care homes are eligible for participation in CACFP, as are for-profit programs that provide care to at least 25% children from families who meet the United States Department of Agriculture (USDA) income eligibility guidelines [22].

The CACFP nutrition standards were revised in 2017 to require participants to serve a greater variety of fruits and vegetables, less solid fats and added sugars, and more whole grains [24]. Results from a national survey conducted shortly before the implementation of these new standards showed that the majority of CACFP center directors reported being ready to comply with the standards [25]. One explanation for this reported readiness is that CACFP participants may have already complied with the new rules prior to their implementation [26]. Still, there may be persistent barriers to serving healthier foods in ECE, like food costs [27–30], children’s preferences [27, 30, 31], staff time and knowledge [28, 32, 33], and parent communication [29]. More research on barriers is needed to better implement healthy eating policies in ECE [34], and studying directors’ perceptions of barriers to serving healthier foods is vital to successful implementation of the updated CACFP standards [29]. Specifically, there have been several recent calls to evaluate how reported barriers differ between CACFP participants and non-participants [35–37]. If CACFP participants experience fewer barriers to providing healthier foods, states seeking to improve the nutrition environment in ECE may be motivated to require all licensed ECE centers and home to comply with CACFP rules, regardless of participation status [35].

The study of barriers to serving healthier foods is one of several factors that characterize the food and nutrition environment in ECE, which also includes what foods are currently served [38] and consumed [39], and how provider mealtime practices, like eating with children, impact the nutrition of children in care [40, 41]. Prior research has established a correlation between CACFP participation and improved nutrition among young children in care, but there is still considerable room for improvement [35, 42, 43]. Less is known about directors’ perceived barriers to serving healthier foods, and how participation in CACFP may impact these barriers. There is limited evidence to suggest that CACFP participants experience fewer barriers to providing healthier foods in ECE [30, 36, 44], but findings from these studies were geographically limited to a single state [30, 36, 44], did not include adjustment for potential confounders in analyses [30, 36], or had a small sample size [44]. These prior studies provide a limited understanding of how participation in CACFP impacts barriers to serving healthier foods in child care centers. Evidence of differences in barriers by CACFP participation status derived from unadjusted analyses do not account for center-level characteristics that may impact a director’s ability to provide healthier foods. Likewise, evidence derived from studies within one state may fail to account for state-level differences in nutrition policies and practices that may impact a director’s ability to provide healthier foods in their centers [8, 33]. It is therefore important to study differences in barriers to serving healthier foods by CACFP participation status across multiple states, adjusting for potential confounders. To the authors’ knowledge, no such study has done so either before or after the implementation of the new CACFP nutrition standards, which were designed to facilitate healthier eating in an accessible, cost-neutral way [45]. Therefore, the purpose of this study was to evaluate barriers to serving healthier foods in child care centers across four states, comparing centers that did and did not participate in CACFP prior to the new CACFP rules taking effect. We hypothesized that more directors participating in CACFP would report experiencing no barriers to serving healthier foods, compared to non-participants. Based on results from prior studies, we also hypothesized that among the entire sample, cost would emerge as a top barrier.

## Methods

### Overview

This study analyzed cross-sectional survey data from a sample of child care center directors in Massachusetts, North Carolina, Rhode Island, and South Carolina. We compiled publicly available lists of all currently-operating, licensed ECE centers with no open case of abuse or neglect in each state (*n* = 9567) and identified 20% of all directors from each state, respectively, to contact using a random number generator. At the time of data collection, members of the study team lived in two of the four states (Massachusetts, North Carolina), and chose two additional neighboring states (Rhode Island, South Carolina) for inclusion. We mailed a total of 1983 surveys, instructions, and stamped return envelopes to directors in 2012. Directors indicated their consent to participate by completing and returning surveys. Directors who returned surveys were entered into a drawing to win gift cards. The Institutional Review Boards of Harvard Medical School and Harvard Pilgrim Health Care and Duke University Medical Center approved this study.

### Survey

We created a 55-question survey related to nutrition and healthy eating in ECE using questions developed by Whitaker et al. [46], Ammerman et al. [47], and Benjamin et al. [48]. The survey included input from stakeholders, including child care directors, teachers, and administrators, and results from pilot tests with ECE directors in each state. For this study, we analyzed responses to 30 questions related to center-, child-, and director-level demographic characteristics, and two questions related to barriers to serving healthier foods in ECE. We developed the two questions related to barriers to serving healthier foods using language taken directly from the Nutrition and Physical Activity Self-Assessment for Child Care (NAP SACC) [48] and the Study of Healthy Activity and Eating Practices and Environments in Head Start (SHAPES) [46]. The NAP SACC self-assessment has been previously tested for both reliability and validity and psychometric properties of this tool are available elsewhere [48, 49]. In one question, center directors were asked to indicate which barriers, if any, they would experience if they tried to provide healthier foods than those they were currently serving. This question was used from the SHAPES survey [46], and did not include a standardized definition of healthier foods, which is consistent with other studies that measured directors’ perceptions of barriers to serving healthier foods in ECE [29, 30, 36, 50]. Thus, directors may have interpreted “healthier food” differently. The list of barriers included: not enough money; lack of control over foods delivered by the supplier; lack of knowledge regarding how to prepare healthier meals; lack of staff time to prepare healthier foods; children would not like the taste of healthier foods; and lack of parental support for serving healthier foods. Directors were also given the option to indicate that they would not experience any barriers to serving healthier foods, or to write in responses not included in the list. Another question asked directors to specify which barrier, if any, was the most significant challenge to serving healthier foods. Two researchers reviewed all write-in responses and, where possible, recoded those that corresponded to predetermined answer choices. For example, the write-in response “kids don’t like to eat these foods” was recoded as the pre-specified option “children would not like the taste of healthier foods.” The two researchers discussed how to recode the write-in variables and reached consensus after any disagreements. Fewer than 5% of responses needed to be recoded. The survey also included demographic questions on total child enrollment; child race and ethnicity as a percent of total enrollment; center profit status; number of teachers, staff, and classrooms; participation in CACFP; and director demographics.

### Analysis

To summarize center and director characteristics, we calculated means, standard deviations (SD), and percentages. The primary outcome was reporting no barriers to serving healthier foods. For secondary outcomes, we examined each of the barriers separately, as well as the total number of barriers reported. We fit unadjusted and adjusted logistic regression models to estimate the odds of directors reporting no barriers to serving healthier foods, and each of the specific barriers, comparing those participating in CACFP and those not participating. We also fit unadjusted and adjusted Poisson regression models to estimate the incidence rate ratio of the total number of barriers reported, comparing centers that did and did not participate in CACFP. We adjusted for covariates including profit status (for profit versus not-for-profit), total child enrollment (continuous), years in operation (continuous), state, and director education (high school, technical college, university degree, graduate degree/higher). We identified these potential confounders a priori because these characteristics have been shown to differ between CACFP and non-CACFP centers [43] and may impact center directors’ readiness to comply with new healthy eating policies [25]. In a prior study, we found similar differences between demographic characteristics and reported barriers to complying with a new healthy eating policy in ECE [30]. We conducted all analyses using Stata 14.1 (StateCorp LP, College Station, TX) with a significance level of α = 0.05.

## Results

We received 713 surveys (response rate of 36%), of which 81 were from Massachusetts (11.4%), 57 from Rhode Island (8.0%), 314 from North Carolina (44.0%), and 261 from South Carolina (36.6%). We excluded 54 surveys for missing or incomplete information germane to this study. Thirty-eight directors (5%) did not explicitly indicate that they served meals or snacks in their center, and 16 directors (2%) did not include their center participation status in CACFP. This resulted in a final sample of 659 centers. At the time of data collection, all states but Rhode Island required licensed child care centers to adhere to CACFP nutrition rules, regardless of their participation status in the program. State-level participation in CACFP varied between study locations, with approximately 26% participation among all child care centers in Massachusetts, 47% in Rhode Island, 44% in North Carolina, 25% in South Carolina, suggesting more variability within, rather than between, regions. All states had quality rating and improvement systems available to center directors, which provided best practice recommendations and guidelines for improving health and quality of care in child care [51]. We did not measure differences in tuition data or urban versus rural location between centers in this study.

Among all directors, 362 centers participated in CACFP (55%) and 331 did not (45%) (Table 1). Nearly all (96%) directors were female. At the time of data collection, directors from CACFP centers reported working in ECE for a mean (SD) 18.2 (9.2) years, and directors from non-CACFP centers reported working in ECE for a mean (SD) 19.1 (9.8) years. There were some demographic differences between CACFP and non-CACFP centers. Non-CACFP center directors reported a higher percentage of white children (66% vs. 35%; *p* < 0.001), and a lower percentage of black children (22% vs. 46%; *p* < 0.001) enrolled in care. A higher percentage of non-CACFP directors reported having a four-year university degree or higher, compared to directors from CACFP centers (64% vs. 56%; *p* < 0.0001). There were no significant differences between CACFP and non-CACFP centers in total child enrollment, number of teachers, staff, or classrooms, or director years of experience.

**Table 1:** Characteristics of centers, directors, and children in Massachusetts, North Carolina, Rhode Island, South Carolina, 2012 (*n* = 659)

	Centers participating in CACFP (*n* = 362)	Centers not participating in CACFP (*n* = 297)
**Center characteristics**	**Mean (SD)**	
Number of children enrolled	64.0 (49.7)	64.6 (47.1)
Years in operation	18.0 (12.9)	20.4 (15.4)
Number of paid staff	13.0 (9.9)	13.2 (11.9)
Number of teachers	9.8 (7.5)	10.8 (10.8)
Number of classrooms	5.4 (3.0)	5.4 (3.5)
	**Number (%)**	
For-profit	208 (58)	192 (65)
**Child characteristics**	**Mean (SD)**	
% Black/African American	45.8 (37.7)	21.7 (31.2)
% White	34.8 (34.9)	65.9 (34.8)
% Hispanic/Latino (a)	7.6 (13.5)	3.7 (10.7)
% Multiple/more than one race	5.4 (10.2)	3.4 (5.3)
% Asian/Asian American	1.6 (6.6)	2.8 (8.8)
% Native American/American Indian	0.9 (6.6)	0.2 (1.0)
**Director characteristics**	**Mean (SD)**	
Years of experience	18.2 (9.2)	19.1 (9.8)
	**Number (%)**	
4-year college degree or higher	200 (56)	183 (64)
Gender, female	342 (96)	276 (96)
**State**		
Massachusetts	6 (2)	69 (23)
Rhode Island	22 (6)	32 (11)
North Carolina	220 (61)	75 (25)
South Carolina	114 (31)	121 (41)

*CACFP* Child and Adult Care Food Program

Overall, 39.5% of center directors reported no barriers to serving healthier foods to children (Table 2). Among directors from centers participating in CACFP, 42.9% reported no barriers, compared to 35.1% of non-CACFP directors who reported no barriers. The most prevalent reported barriers to serving healthier foods in child care centers were cost (41.9%) and children’s food preferences (19.4%). Cost was reported as a barrier by 39.7% of CACFP directors and 48.6% of non-CACFP directors. Children’s food preferences was reported as a barrier by 22.4% of CACFP directors and 17.5% of non-CACFP directors. Among all directors who indicated a top barrier (exclusive of all other barriers), 56.9% of CACFP directors and 66.3% of non-CACFP directors reported cost as the top barrier. Fewer than 10% of directors reported any other barrier (other than cost and children’s food preferences) to serving healthier foods in centers.

**Table 2:** Unadjusted analyses examining barriers to serving healthier foods in ECE in CACFP and non-CACFP centers, *n* = 659

	Centers participating in CACFP (*n* = 362)	Centers not participating in CACFP (*n* = 297)
	Frequency (%)	
**No barriers**	151 (42.9)	98 (35.1)
**Barriers to serving healthier foods**		
Not enough money to serve healthier meals and snacks	140 (39.7)	136 (48.6)
Children do not like the taste of healthier meals and snacks	79 (22.4)	49 (17.5)
Staff do not have time to prepare healthier meals and snacks	29 (8.2)	24 (8.6)
Lack of control over what is delivered by food service provider	28 (7.9)	24 (8.6)
Parents do not support the idea of serving children healthier meals and snacks	26 (7.4)	19 (6.8)
Staff lack the knowledge to prepare healthier meals and snacks	16 (4.5)	16 (5.7)
**Cost as top barrier**	111 (56.9)	112 (66.3)
	Mean (SD)	
**Total number of barriers reported**	0.93 (0.99)	1.04 (0.99)

After adjusting for profit status, total child enrollment, years in operation, state, and director education, directors from CACFP centers had more than twice the odd of reporting no barriers to serving healthier foods in child care centers, compared to directors from non-CACFP centers (OR = 2.03, 95% CI [1.36, 3.04]; *p* = 0.001) (Table 3). Directors from CACFP centers were less likely to report cost as a barrier (OR = 0.46; 95% CI [0.31, 0.67]; *p* < 0.001), and less likely to report cost as the top barrier (OR = 0.48; 95% CI [0.28, 0.81]; *p* = 0.007), compared to directors from non-CACFP centers. Finally, CACFP directors reported fewer barriers overall (IRR = 0.77; CI [0.64, 0.92]; *p* < 0.01), compared to non-CACFP directors.

**Table 3:** Adjusted odds ratios (OR) and incidence rate ratios (IRR) in analyses examining barriers to serving healthier foods in ECE in CACFP and non-CACFP centers, *n* = 659

	OR^**a**^ (95% CI)	***P***
**No barriers**	2.03 (1.36–3.04)	0.001
**Barriers to providing healthier foods**		
Not enough money to serve healthier meals and snacks	0.46 (0.31–0.67)	< 0.001
Children do not like the taste of healthier meals and snacks	1.11 (0.70–1.76)	0.65
Staff do not have time to prepare healthier meals and snacks	0.65 (0.33–1.26)	0.20
Lack of control over what is delivered by food service provider	0.78 (0.40–1.58)	0.47
Parents do not support the idea of serving children healthier meals and snacks	1.62 (0.75–3.51)	0.22
Staff lack the knowledge to prepare healthier meals and snacks	0.47 (0.20–1.10)	0.08
**Cost as top barrier**	0.48 (0.28–0.81)	0.007
	**IRR**^**a**^**(95% CI)**	**p**
**Total number of barriers reported**	0.77 (0.64–0.92)	0.005

^a^Adjusted CACFP participation, profit status, total child enrollment, years in operation, state, and director education

## Discussion

In this cross-sectional survey of 659 center directors from four states, we found directors from CACFP centers were more likely to report experiencing no barriers to serving healthier foods in child care centers, compared to directors from non-CACFP centers. Among all centers (both CACFP and non-CACFP), the most prevalent barriers to serving healthier foods were cost and children’s food preferences. However, directors from CACFP centers reported fewer barriers than directors from non-CACFP centers, and were less likely to report cost as a top barrier.

These findings are consistent with our hypotheses and prior research in this area [30, 36, 44]. Studies in Georgia [36], Illinois [44], and South Carolina [30] have reported differences in barriers to serving healthier foods by participation in CACFP, all indicating that participating centers and family child care homes may experience fewer barriers than non-participants. In a cross-sectional survey of child care center directors in South Carolina, Zaltz et al. [30] found fewer CACFP directors reported cost as a barrier to implementing new healthy eating standards, compared to non-participants. In the same study, more CACFP directors reported adherence to several nutrition best practices, like maintaining a written nutrition policy and using non-food items for holidays and other celebrations [30]. Cotwright et al. [36] reported similar findings in a recent statewide survey of ECE directors in Georgia who implemented a new beverage policy. In that study, fewer CACFP directors reported barriers to meeting juice and milk guidelines, and those directors reported serving fewer sugar-sweetened beverages overall, compared to non-CACFP directors [36]. Results from these studies, which collectively suggest that CACFP participants may experience fewer barriers to serving healthier foods and beverages in ECE, are limited by the fact that they report results from unadjusted analyses [30, 36]. The results from this paper build upon this prior work by examining CACFP participation as the main predictor of barriers to serving healthier foods, controlling for important potential confounders like center profit status, size, number of children and staff, or director education.

There are several potential explanations for the association between CACFP participation and fewer reported barriers to serving healthier foods and beverages in child care centers. First, compared to non-participating centers, CACFP centers may already serve healthier foods [35, 42, 43]. Ritchie et al. [42] compared foods served in California ECE centers and family child care homes, including those that did and did not participate in CACFP, using a self-reported food frequency checklist. Compared to non-CACFP participants, CACFP centers and family child care homes served more milk, more vegetables, and fewer sugar-sweetened beverages [42]. Korenman et al. [43] also reported increased servings of milk and vegetables among CACFP participants, using nationally representative food frequency data collected as part of the Early Childhood Longitudinal Study, Birth Cohort [43, 52]. Finally, Andreyeva et al. [35] measured plate waste and directly-observed lunchtime intake among a random sample of child care centers in Connecticut and reported differences in nutritional intake by CACFP status. In that study, children at CACFP centers were more likely to consume low-fat milk and less likely to consume saturated and trans fats [35].

Next, CACFP centers may be better equipped to address cost barriers, since they receive reimbursements for serving healthy foods [23]. In this study, cost was the most prevalent barrier reported to serving healthier foods in centers. This finding has also been reported in prior studies [27, 28, 30, 50, 53]. Recently, Nanney and colleagues [50] assessed the implementation of nutrition policies among CACFP and non-CACFP ECE directors in Minnesota and Wisconsin centers and family child care homes, of whom 80% reported cost as a barrier. In our study, more than 40% of ECE directors across four states reported cost as a barrier, including both CACFP and non-CACFP participants. However, directors from centers participating in CACFP were less likely than non-CACFP directors to report cost as a barrier, and less likely to identify cost as the top barrier. Still, some limited evidence from qualitative research suggests that CACFP reimbursements may not be sufficient to overcome the cost burden of serving healthier foods [28]. Future research should assess cost barriers to serving healthier foods among CACFP participants after the implementation of updated nutrition rules in 2017 [45]. These new rules, which required participants to serve a greater variety of fruits, vegetables, and whole grains, were designed to be cost-neutral, and did not include additional reimbursements [45]. There may be reason, however, to increase federal reimbursements to CACFP participants, since cost barriers are consistently reported, and prior evidence has shown that CACFP participants incur additional costs when serving more fruits, vegetables, and whole grains [53]. Increased federal reimbursements for healthier foods may help alleviate the cost barrier, while also improving children’s diet quality. In fact, there is some evidence to suggest that ECE providers who receive more federal food subsidies serve higher nutritional quality foods to children [54].

Finally, CACFP participants may experience fewer barriers to serving healthier foods in child care centers since they receive nutrition trainings [22]. Training in nutrition may help reduce barriers to both identifying and serving healthier foods and beverages [34]. Additionally, CACFP participants have been shown to adhere to supportive nutrition practices associated with healthier eating in ECE, like serving family-style meals [33, 35], having providers eat the same foods as children [15, 35], and providing nutrition education to parents [44]. In a qualitative study of ECE directors from central Illinois, Dev and colleagues [44] found that non-CACFP directors reported more barriers to encouraging parents to provide healthier foods from home, which is a recommended best practice for nutrition in ECE [7]. To improve the potential impact of CACFP on children’s dietary intake, policymakers and researchers should continue to evaluate participation and its potential role in reducing barriers to serving healthier foods and beverages in ECE. If participating centers continue to report fewer barriers to healthier eating, states may wish to require all licensed ECE centers and homes to comply with CACFP standards. Additionally, states may provide non-participants with resources like staff nutrition trainings that may reduce barriers to healthier eating in ECE. Prior findings and results from this study indicate an overall improved nutrition environment among CACFP centers via increased training, capacity, and resources among staff, directors, children, and parents. When considered holistically, these findings suggest that participation in CACFP may itself be an effective strategy to decrease barriers to healthier eating. Future research should thus focus on barriers to participating in CACFP, which may include a variety of factors like administrative capacity of the center, community engagement, pre-existing center policies, and provider/director attitudes [55].

This study is one of a few to specifically evaluate barriers to serving healthier foods in child care centers by CACFP participation status, and the first to do so via adjusted analyses across two regions within the US. However, this study has limitations. First, we could not assess causality due to the cross-sectional nature of these data. We also did not provide a standardized definition of healthier foods to survey respondents, consistent with similar, quantitative survey-based studies [29, 30, 36, 50]. These findings, therefore, reflect directors’ barriers to serving healthier foods couched within their perceptions and beliefs of what constitutes healthier foods. Directors may have interpreted “healthier food” differently. Qualitative research on directors’ perceptions of healthier eating in ECE can help bridge this gap [44, 56], and there have been recent calls for more research on factors associated with barriers to healthier eating in CACFP centers [29]. Second, generalizability of these results is limited by a 36% response rate. However, our response rate is comparable to those reported in similar studies in ECE [30, 42, 57], and demographic characteristics of CACFP and non-CACFP centers in this study are similar to those from two nationally-representative surveys of child care centers [25, 43]. Third, we surveyed center directors, who are well-positioned to report barriers, because they are likely to be involved in the procurement of foods and beverages served to children [58]. But, we did not survey teachers or other staff members in the centers, who may provide unique perspectives on the barriers to healthy eating in ECE because they may be more involved with the planning, preparation, and serving of foods to children [59]. Teachers and directors may also perceive barriers to healthier eating differently based on varying values or priorities related to improving nutrition in ECE. Fourth, we did not assess the nutritional quality of foods and beverages served to children in these centers, so we are not able to connect reported barriers to actual nutritional content of foods served. Prior studies suggest, however, that meals and snacks served in ECE are often lacking in vitamin A, iron, and folate [60–62], and one study conducted in North Carolina found child care center providers to serve an inadequate amount of fruits, vegetables, and whole grains [63]. Thus, there is some evidence that centers have room for improvement in the nutritional quality of foods and beverages served. Finally, social desirability bias may have influenced our findings, as directors may be more likely to report practices that reflect favorably on their centers. On the other hand, directors who responded to the survey may be more likely to report more barriers compared to non-respondents, if their initial participation in the study was influenced by a desire to communicate challenges in ECE.

## Conclusions

This study presents results from cross-sectional survey data related to child care center director-reported barriers to serving healthier foods to children. Findings from this study suggest that directors participating in CACFP were less likely to report barriers to serving healthier foods, compared to non-CACFP directors. These findings were derived from a random sample of child care center directors across four US states, which represents the largest and most geographically diverse study to examine the impact of CACFP participation on barriers to serving healthier foods. Examining barriers to serving healthier foods across multiple states is important, considering state and regional differences in policies and practices among ECE centers [25, 43, 64]. While barriers to serving healthier foods are just one component of supportive nutrition environments within ECE, they serve as an important indicator of readiness to implement new healthy eating policies [34, 65]. Findings from this study add to the growing body of literature related to CACFP participation, healthy eating environments, and readiness to implement new nutrition-related policies in ECE. This assessment is of particular importance, since no baseline data exist to examine the impact of CACFP participation on barriers to serving healthier foods in child care centers prior to rule changes in 2017. Future research should re-evaluate barriers to healthier eating in CACFP centers after the rule changes, to assess whether common barriers persist.

## Data Availability

The data used during this study are available from the corresponding author on reasonable request.

## Abbreviations

CACFP: Child and Adult Care Food Program
ECE: Early care and education
USDA: United States Department of Agriculture
NAP SACC: Nutrition and Physical Activity Self-Assessment for Child Care
SHAPES: Study of Healthy Activity and Eating Practices and Environments in Head Start
